# Increased peripheral blood inflammatory cytokine levels in amyotrophic lateral sclerosis: a meta-analysis study

**DOI:** 10.1038/s41598-017-09097-1

**Published:** 2017-08-22

**Authors:** Yang Hu, Chang Cao, Xiao-Yan Qin, Yun Yu, Jing Yuan, Yu Zhao, Yong Cheng

**Affiliations:** Center on Translational Neuroscience, College of Life and Environmental Sciences, Minzu University of China, Beijing, 100081 China

## Abstract

Amyotrophic lateral sclerosis (ALS) is a fatal neurodegenerative disease with poorly understood etiology. Increasing evidence suggest that inflammation may play a critical role in the pathogenesis of ALS. Several studies have demonstrated altered levels of blood cytokines in ALS, but results were inconsistent. Therefore, we did a systematic review of studies comparing blood inflammatory cytokines between ALS patients and control subjects, and quantitatively combined the clinical data with a meta-analysis. The systematic review of Pubmed and Web of Science identified 25 studies encompassing 812 ALS patients and 639 control subjects. Random-effects meta-analysis demonstrated that blood tumor necrosis factor-α (TNF; Hedges’ g = 0.655; p = 0.001), TNF receptor 1 (Hedges’ g = 0.741; p < 0.001), interleukin 6 (IL-6; Hedges’ g = 0.25; p = 0.005), IL-1β (Hedges’ g = 0.296; p = 0.038), IL-8 (Hedges’ g = 0.449; p < 0.001) and vascular endothelial growth factor (Hedges’ g = 0.891; p = 0.003) levels were significantly elevated in patients with ALS compared with control subjects. These results substantially enhance our knowledge of the inflammatory response in ALS, and peripheral blood inflammatory cytokines may be used as diagnostic biomarkers for ALS in the future.

## Introduction

Amyotrophic lateral sclerosis (ALS), also known as Lou Gehrig’s disease, is a fatal neurodegenerative disease characterized by the degeneration of motor neurons in brain and spinal cord^1^. The loss of motor neuros in ALS patients usually cause death within 2–5 years after diagnosis due to respiratory failure^2^. The prevalence of the devastating disease is unknow for much of the world, and a prevalence of 3.9 cases of ALS per 100, 000 persons in the U.S was found during October 2010 to December 2011^3^. Although 5–10% cases identified as familial ALS which linked to genetic mutations, 90–95% cases being sporadic with the cause remains unknown^4^. There is no cure for ALS, the only drug approved by FDA is riluzole which was found to modestly extend life by approximately two to three months^5^. Therefore, there is an urgent need to better understand the etiology of ALS and subsequently develop more effective therapy for the devastating disease.

Increasing evidence suggest that inflammatory response in the central nervous system (CNS) that includes proinflammatory cytokines contribute to the pathogenesis of ALS^6^. Neuroinflammation has been identified as a prominent pathological signature in ALS, both post-mortem and PET imaging studies revealed microglial activation in the patients with ALS^7, 8^. Moreover, infiltration of immune cells have been found in the CNS of ALS patients, these include macrophages and T-cells at sites of motor neuron injury^9–11^.

In addition, clinical studies have also suggested that peripheral inflammation is implicated in ALS, and the peripheral immune abnormalities include T-cells, cytokines, chemokines and other markers of inflammation^11^. The easy access to blood have led to more and more researchers to study peripheral blood cytokine and chemokine aberrations in ALS, in hope of gaining novel insights into the pathogenesis of ALS and potential early diagnosis of the disease, and even disease modifying treatments. A number of studies have demonstrated elevated blood levels of inflammatory cytokines and chemokines in patient with ALS, such as tumor necrosis factor-α (TNF-α), interleukin-6 (IL-6) and monocyte chemotactic protein 1 (MCP-1)^12–15^. However, other studies suggested that the levels of cytokines in ALS patients were unchanged compared to control subjects^16–18^. To address the inconsistent results from clinical studies, we aim to search the literature systematically and quantitatively summarize the clinical data comparing blood cytokine levels between ALS patients and control subjects.

## Methods

This systematic review and meta-analysis was performed according to the guidelines that are recommended by the PRISMA statement (Preferred Reporting Items for Systematic reviews and Meta-Analysis)^19^.

### Search strategy and study selection

Two independent researchers (Y.H. and C.C.) performed a systematic review of peer-reviewed English-language articles from Pubmed and Web of Science. The database search term was: (inflammation or cytokine or chemokine or interleukin or tumor necrosis factor or interferon) AND (Amyotrophic lateral sclerosis or ALS). The search date started on May 11, 2016 and ended on January 1, 2017. Original studies that comparing peripheral blood cytokines between patients with ALS and control subjects were included, and we undertook meta-analysis whenever individual inflammatory cytokine data were available in three or more articles.

### Data extraction

Data were extracted by two of us (Y.H. and C.C.). We extracted data on mean cytokine concentrations, standard deviation, p value and sample size to calculate effective size (ES) for meta-analysis. We also extracted data on age, sex, disease duration, control type and sampling source for potential between-study heterogeneity analysis (Supplementary Table).

### Statistical analysis

Comprehensive Meta-analysis software (version 2; Biostat Inc) was used to perform all the statistical analyses in this study. ESs were generated from sample size and mean cytokine concentrations with standard deviation, or sample sizes and P values. In some studies, P values were reported as inequality rather than exact values, or reported as no significant differences of cytokine concentrations between ALS patients and control subjects. We then contacted the corresponding authors of the original articles to request the necessary data to generate effective sizes. When the data were not available from the original articles or corresponding authors, we calculated the ESs as described previously^20, 21^. ESs were calculated as the standardized differences in mean cytokine concentrations between ALS patients and controls, and then converted to Hedges *g* statistic, which adjusted the ESs based on sample sizes^22^. An ES estimate was calculated for individual cytokine whenever the cytokine data were reported in at least three studies. A random-effects model was undertaken for the meta-analysis as we hypothesized that both within-study and between-study heterogeneity modulated the true ES^23^. Sensitivity analysis was undertaken to test the robustness of the outcome of the meta-analysis, this was achieved by excluding one study at a time to perform meta-analysis.

The I^2^ statistic and Cochrane Q test were used to analyze the between-study heterogeneity as described previously^23^. I^2^ statistic of 0.25, 0.5, 075 indicated small, moderate, and high levels of variance among studies. Unrestricted maximum-likelihood random-effects meta-regressions of ES^24^ were performed to test whether sample size, patient age and gender (proportion of male individuals), and disease duration had moderating effects on the outcomes of the meta-analysis. Publication bias was determined by the Egger test as described previously^25^. All values for significances in this study were set at *P* < 0.05 except statistical difference for the Cochrane Q test, which was set at *P* < 0.10.

## Results

**Figure 1:**
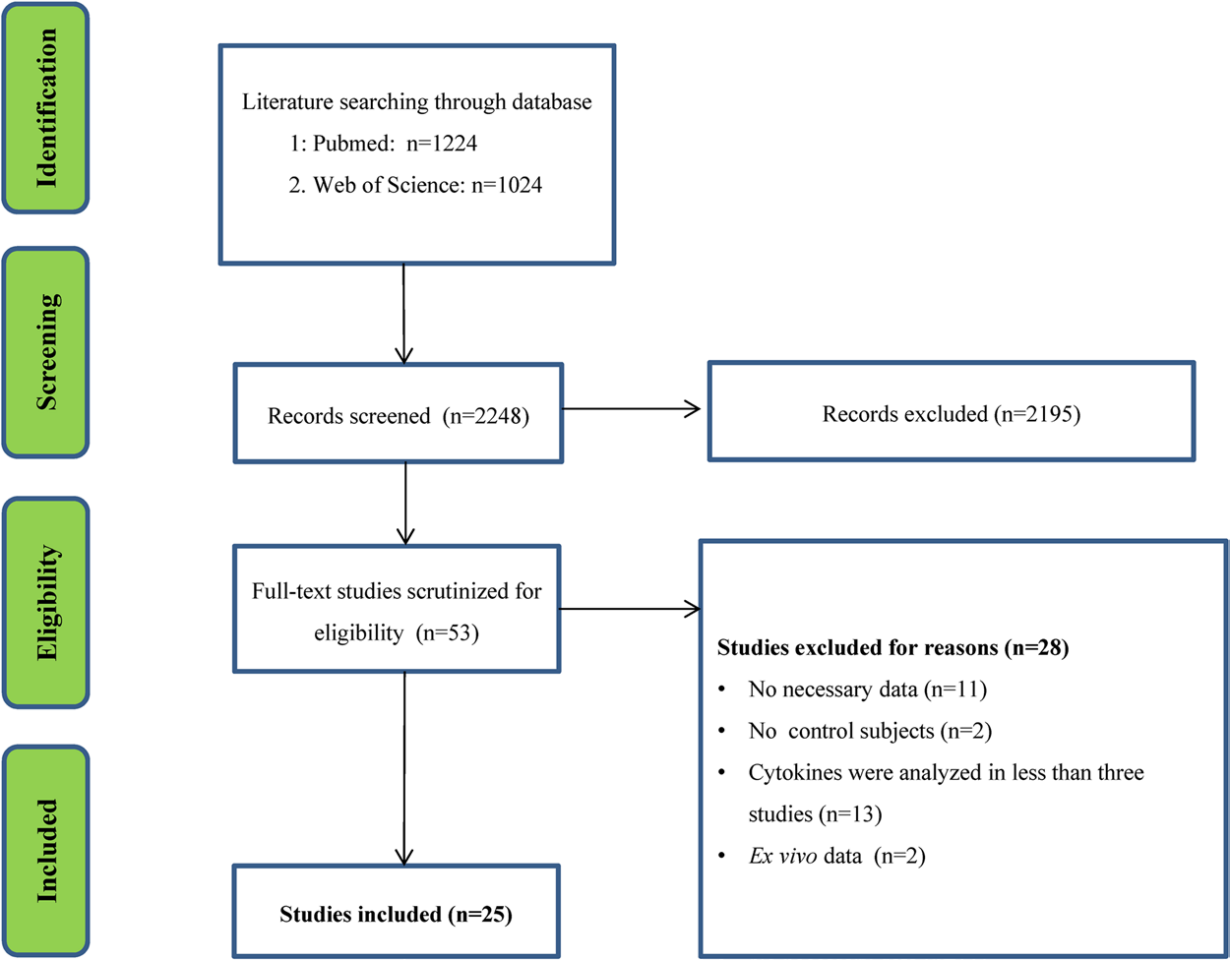
PRISMA flowchart of the literature search.

The systematic review of the literature identified 1224 records from PubMed and 1024 from Web of Science. After screening the titles and abstracts, 53 articles were selected for full text review. 28 studies were excluded because they reported individual cytokine data in less than three studies (13 studies), lacked necessary data (11 studies), lacked a control group (2 studies), reported cytokine levels *in vitro* (2 studies). Therefore, 25 articles comparing 14 blood inflammatory cytokines between 812 ALS patients and 639 controls were included in this meta-analysis^12–18, 26–43^ (Fig. 1).

### Main association of ALS with blood cytokine levels

**Figure 2:**
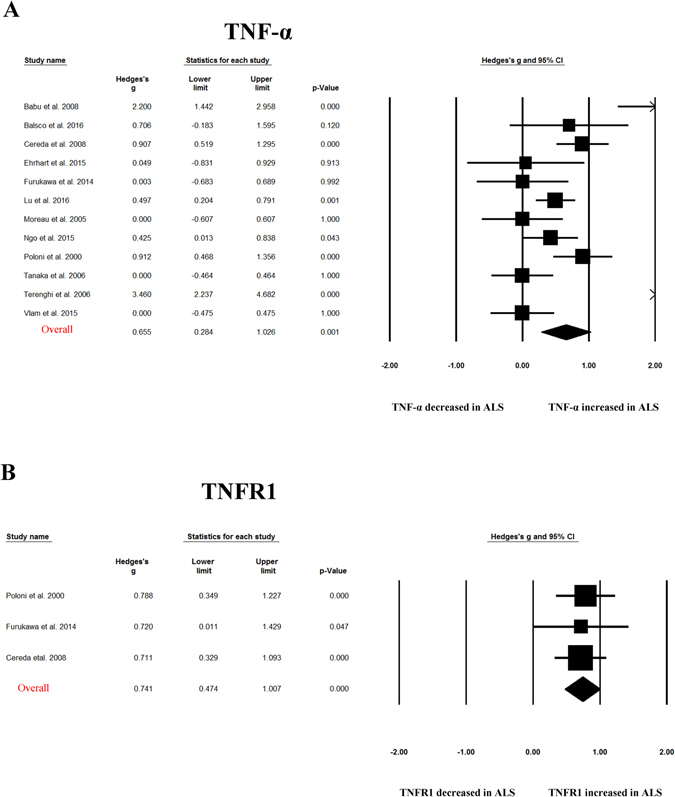
Studies of peripheral blood TNF-α and TNFR1. Forest plot displaying random effects meta-analysis results of the association between TNF- α **(A)**, TNFR1 **(B)** and ALS. The sizes of the squares are proportional to study weights.

**Figure 3:**
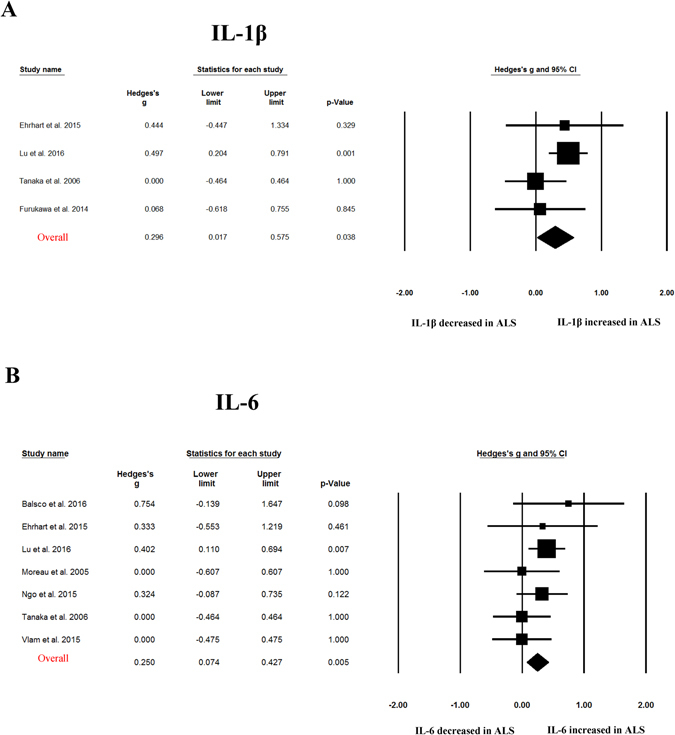
Studies of peripheral blood IL-1β and IL-6. Forest plot displaying random effects meta-analysis results of the association between IL-1β **(A)**, IL-6 **(B)** and ALS. The sizes of the squares are proportional to study weights.

**Figure 4:**
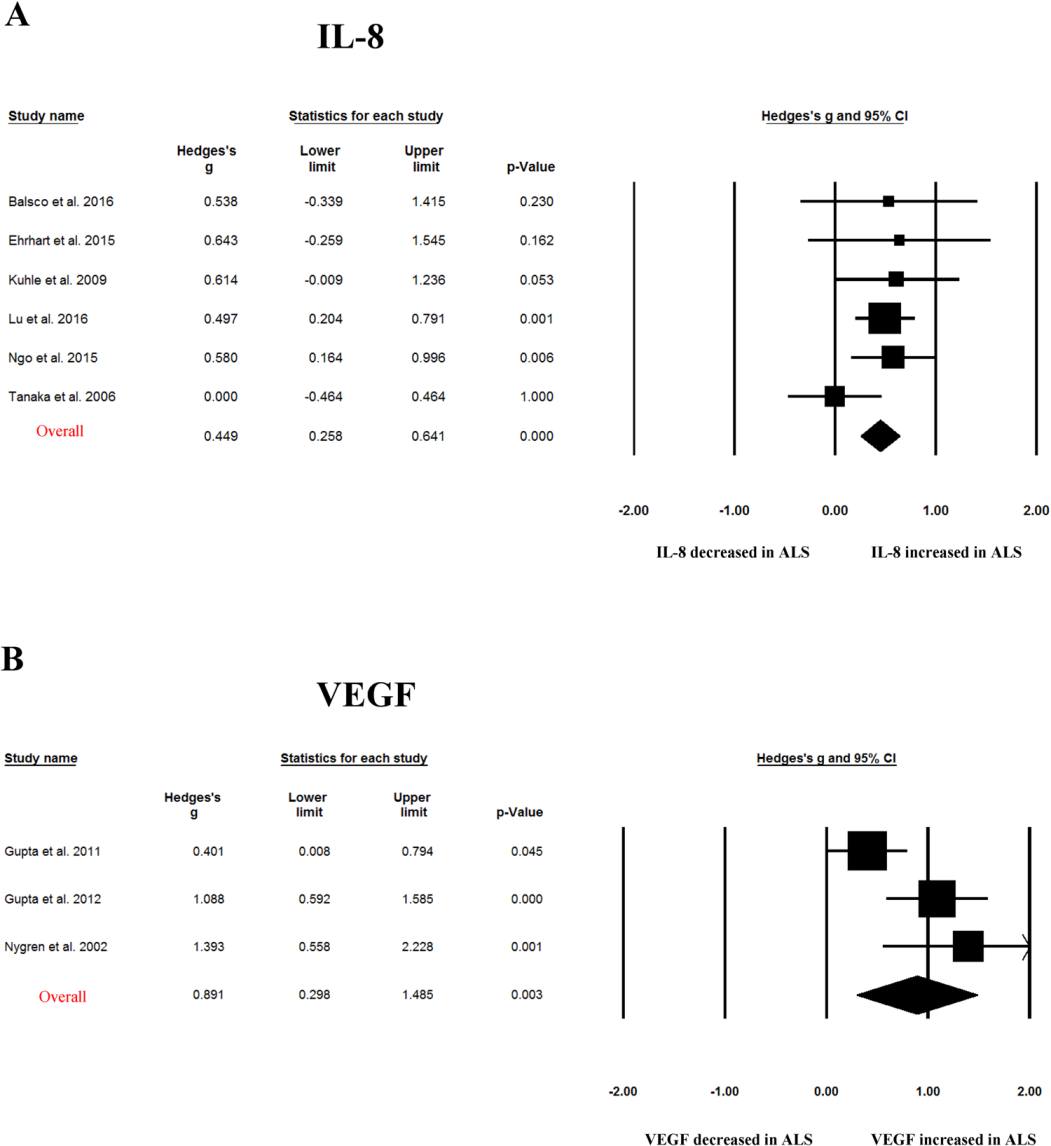
Studies of peripheral blood IL-8 and VEGF. Forest plot displaying random effects meta-analysis results of the association between IL-8 **(A)**, VEGF **(B)** and ALS. The sizes of the squares are proportional to study weights.

Random-effects meta-analysis demonstrated that blood TNF-α (Hedges’ g = 0.655; 95% CI, 0.284 to 1.026; p = 0.001), TNF receptor 1 (TNFR1; Hedges’ g = 0.741; 95% CI, 0.474 to 1.007, p < 0.001), IL-6 (Hedges’ g = 0.25; 95% CI, 0.074 to 0.427; p = 0.005), IL-1β (Hedges’ g = 0.296; 95% CI, 0.017 to 0.575; p = 0.038), IL-8 (Hedges’ g = 0.449; 95% CI, 0.258 to 0.641; p < 0.001) and vascular endothelial growth factor (VEGF; Hedges’ g = 0.891, 95% CI: 0.298, 1.485; p = 0.003) levels were significantly elevated in patients with ALS compared with control subjects (Figs 2–4 and Table 1). In contrast, blood IL2, IL-4, IL-5, IL10, IL17, IFN-gamma, endothelial leukocyte adhesion molecule (ELAM-1), monocyte chemotactic protein-1 (MCP-1) levels did not show significant differences between ALS patients and controls (Table 1)

**Table 1:** Summary of comparative outcomes for peripheral blood cytokine measurements.

Cytokine	No. of studies	No. with ALS/ Controls	main effects			Heterogeneity				Publication Bias	
			Hedges g (95% CI)	z Score	P Value	Q Statistic	df	P Value	I^2^ Statistic	Egger Intercept	P Value
TNF-α	12	456/366	0.655 (0.284 to 1.026)	3.462	0.001	63.726	11	0	82.739	1.98	0.33
MCP-1	8	317/245	0.351 (−0.100 to 0.801)	1.526	0.127	33.374	6	0	82.022	3.00	0.60
IL6	7	267/242	0.250 (0.074 to 0.427)	2.776	0.005	5.252	6	0.512	0	−0.28	0.81
IL8	6	242/192	0.449 (0.258 to 0.641)	4.591	<0.001	4.566	5	0.471	0	0.20	0.87
IL2	5	186/193	0.130 (−0.239 to 0.498)	0.689	0.491	10.350	4	0.035	61.353	−2.84	0.17
IFN-r	4	206/172	0.643 (−0.418 to 1.703)	1.188	0.235	64.651	3	0	95.36	11.71	0.11
IL1-beta	4	161/143	0.296 (0.017 to 0.575)	2.076	0.038	3.816	3	0.282	21.374	−1.24	0.50
IL10	4	154/138	0.305 (−0.038 to 0.648)	1.742	0.081	3.166	2	0.205	36.823	−1.52	0.65
IL4	3	145/128	0.069 (−0.496 to 0.634)	0.241	0.81	7.642	2	0.022	73.828	−4.00	0.14
IL5	3	145/128	0.042 (−0.552 to 0.635)	0.138	0.89	8.395	2	0.015	76.178	−4.20	0.12
IL17	3	102/62	0.550 (−0.079 to 1.178)	1.713	0.087	7.009	2	0.03	71.467	7.9 0	0.25
TNFR1	3	155/91	0.741 (0.474 to 1.007)	5.442	<0.001	0.071	2	0.965	0	0.01	1.00
ELAM-1	3	51/42	0.301 (−0.101 to 0.703)	1.468	0.142	1.132	2	0.568	0	3.25	0.83
VEGF	3	107/92	0.891 (0.298 to 1.485)	2.943	0.003	7.091	2	0.029	71.796	4.64	0.37

N, sample size; CI, confidence interval; ALS, amyotrophic lateral sclerosis; TNF, tumor necrosis factor-α; TNFR1, TNF receptor 1; IFN, interferon; IL, interleukin; ELAM, endothelial leukocyte adhesion molecule; MCP-1, Monocyte chemotactic protein-1; VEGF, vascular endothelial growth factor; df, degree of freedom; Q, Cochran’s Q test; z (test of null hypothesis); p (statistical significance); I^2^ (heterogeneity level).

### Investigation of heterogeneity

This meta-analysis found significant heterogeneity for 8 of 14 cytokines. IL-2, IL-4, IL-17, VEGF showed moderate levels of heterogeneity, whereas TNF-α, MCP-1, IFN-gamma, IL-5 showed high levels of heterogeneity (Table 1). Next, we attempted to explore the moderators that explained the heterogeneity among studies in the meta-analysis. These potential moderators include categorical variables (sample source and control type) and continuous variables (age, gender, sample size, disease duration). As shown in the eTable in the supplement, the number of studies were limited for most of the cytokines with significant between-study heterogeneity. In addition, only 4 of 25 studies included in this meta-analysis analyzed plasma cytokine levels. We therefore performed sub-group (based on control type) and meta-regression (based on age, gender, sample size and disease duration) analyses for TNF-α.

Sub-group analyses revealed that blood TNF-α levels were significantly increased in ALS patients compared with normal control subjects (Supplementary Figure 1, Hedges’ g = 0.972; 95% CI, 0.43 to 1.514; p < 0.001), and the high levels of heterogeneity remained for normal control group (Q_7_ = 48.459; P < 0.001; I^2^ = 85.555). In contrast, no significant difference was found between ALS patients and disease control subjects (Hedges’ g = 0.201; 95% CI, −0.106 to 0.509; p = 0.2) for blood TNF-α levels, and the disease control group did not show significant between-study heterogeneity (Q_3_ = 4.949; P = 0.176; I^2^ = 39.377).

Meta-regression analyses suggested that gender, disease duration and sample size had no moderating effects on the outcome of the meta-analysis for studies measuring blood TNF-α levels (supplementary Figure 2). However, meta-regression on age showed a significant association between age and ES for studies analyzing TNF-α (Supplementary Figure 2A) (regression coefficient [SE], −0.056 [0.018]; 95% CI, −0.093 to −0.020; *P* = 0.002), suggesting that age had moderating effects on the outcome the meta-analysis.

Sensitivity analyses demonstrated that no individual study significantly influenced the statistically significant differences in blood TNF-α, TNF-R1 and IL-8 levels between ALS patients and control subjects. In contrast, we found that a single study could influence the statistically significant differences in blood IL-1β, IL-6 and VEGF levels between ALS patients and control subjects.

Results from the Egger test suggested that no significant risk for publication bias for cytokines analyzed in the meta-analysis (Egger intercept range, −4.20 to 11.71; *P* > 0.10 in all analyses) (Table 1).

## Discussion

This meta-analysis included 25 case-control studies assessing 812 ALS patients and 639 control subjects, and found evidence of significant elevations of peripheral blood inflammatory cytokines for TNF-α, TNFR1, IL1β, IL-6, IL-8 and VEGF in ALS patients compared with controls. For those cytokines significantly associated with ALS, medium to large ESs were found for TNF-α, TNFR1 and VEGF, whereas IL1β, IL-6, IL-8 showed small to medium ESs. Sensitivity analyses suggested that the significant associations between blood TNF-α, TNFR1 or IL-8 levels and ALS were not influenced by an individual study, suggesting the robustness of these associations. However, the statistically significant associations observed in this meta-analysis for cytokines IL1β, IL-6 and VEGF could be influenced by a single study. This is due to the small ESs associated the results for IL1β and IL-6, and the limited number of studies for VEGF (3 studies).

Neuroinflammation is a prominent feature in neurodegenerative diseases such as Alzheimer’s disease (AD), Parkinson’s disease (PD), and ALS which are characterized by the appearance of reactive microglial and astroglial cells in central nervous system^44^. Moreover, a substantial number of studies suggested that peripheral inflammation may also contribute to the pathological mechanisms of neurodegenerative diseases, with interests on the aberrant levels of peripheral blood inflammatory cytokines in patients with AD, PD and ALS^11, 45^. Previous meta-analyses have been performed for peripheral blood cytokine levels in AD and PD. Similar to the findings of our present meta-analysis in ALS, levels of TNF-α, IL-6 and IL-1β were elevated in patients with AD and PD^46–48^, suggesting that the elevations of blood inflammatory cytokine TNF-α, IL-6 and IL-1β are not specific in ALS. Furthermore, the increased concentrations of blood TNFR1 in ALS patients found in this meta-analysis may also not be specific in ALS, because two studies have consistently demonstrated that patients with PD had increased levels of TNFR1 compared with control subjects^49, 50^. However, the inflammatory cytokine IL-8, which the present meta-analysis showed significant association with ALS from 6 studies, and which zero heterogeneity was found among studies, was not associated with AD or PD in the respective meta-analyses^47, 48^. Another inflammatory cytokine VEGF, which the meta-analysis identified as the cytokine with largest ES in ALS studies, and which is also well known for its roles in angiogenesis and neuroprotection ^51^, may be specifically elevated in ALS. This is due to the two studies which showed no association between blood VEGF and PD^52, 53^, and our unpublished meta-analysis suggests that blood VEGF is not significantly associated with AD. These above results indicate that major neurodegenerative diseases including AD, PD and ALS may have both shared and distinct inflammatory responses.

A biomarker was defined as “a characteristic that is objectively measured and evaluated as an indicator of normal biological processes, pathogenic processes, or pharmacologic responses to a therapeutic intervention.” by the National Institutes of Health Biomarkers Definitions Working Group^54^. Biomarker research from blood have conducted intensively over the past two decades in neurodegenerative diseases including ALS, as it is non-invasive, easy to access and handle with low cost. ALS is diagnosed based on clinical evaluations and can be easy identified at its full-blown presentation. However, there is a pronounced delay between the onset of symptoms and diagnosis, with the diagnostic process as long as between 13 to 18 months^55^. Although only one drug (riluzole) with modest disease-modifying potency is available for ALS^4^, it is generally accepted that the more effective therapeutic interventions with earlier diagnosis. In addition, the lack of validated biomarkers in disease progression and patient prognosis of ALS may contribute to the failed effort for clinical trials over the last half century. Therefore, there is an urgent need to find molecular biomarkers that could give reliable information on the onset and/or progression of ALS. Peripheral blood inflammatory cytokine would be of particularly attractive biomarkers, because there are multiple assays available for peripheral cytokines, and potentially additional mechanistic research to better understand the role of inflammation in the devastating disease and subsequent therapeutic interventions. Indeed, clinical results from different groups suggested that the inflammatory cytokines are promising biomarkers for ALS, with a variety number of studies showed alterations of blood inflammatory cytokines in patients with ALS when compared with controls^12–15^. In addition, one study showed that serum MCP-1 protein, MCP-1 mRNA, VEGF mRNA, smoking and alcohol consumption are the independent variables that differentiated ALS and controls, with a sensitivity of 93.2% and specificity of 86.2%. However, the alterations of cytokines were inconsistent for individual cytokines and between studies^15–17, 29^. In this meta-analysis, we for the first time showed consistent aberrations of several cytokines in ALS from published literature. Concentrations of peripheral blood TNF-α, TNFR1, IL-6, IL-1β, IL-8, VEGF were significantly higher in patients with ALS when compared with controls. For those cytokines significantly associated with ALS, the ESs associated with the results of TNF-α, TNFR1 and VEGF were large, suggesting the potential usefulness of these cytokines as practical diagnostic biomarkers for ALS in the future. Additionally, these potential inflammatory cytokine biomarkers from blood may be used to better estimate the rate of ALS progression and stratify patients with ALS in clinical trials. In fact, Babu *et al*. found that blood TNF-α levels are positively correlated with disease duration of ALS^12^. Lu *et al*. followed a cohort of ALS patients longitudinally and demonstrated IL-6 had significantly increased expression towards end-stage disease in the longitudinal analysis^15^.

The heterogeneity for the individual cytokines in our present meta-analysis varied from zero to high. For the cytokines significantly associated with ALS, high and moderate levels of heterogeneity were found for TNF-α and VEGF studies, respectively, whereas studies for TNFR1, IL1β, IL-6 and IL-8 did not show significant heterogeneity in this meta-analysis. The strength of this study is that meta-analytic technique with subgroup and meta-regression analyses were used to adjust for the potential confounders that explained the high level between-study heterogeneity for TNF-α, although the limited number of studies for VEGF prevented us to further analyze the heterogeneity. Subgroup analysis stratified by control type suggested that the ES and statistical significance were increased for TNF-α studies when compared ALS patients with normal controls, whereas TNF-α levels were not significantly elevated in patients with ALS when compared with disease controls, although the heterogeneity remained high for studies comparing ALS patients and normal controls. This is reasonable as we have discussed above that the alterations of TNF-α levels in ALS may be not specific in neurodegenerative diseases, and most disease control subjects in this meta-analysis were neurological disease. Meta-regression analysis demonstrated that age had moderating effect on the outcome of the meta-analysis, whereas other potential confounders including gender, disease duration and samples size did not have moderating effects on the outcome of the meta-analysis, suggesting that the moderating effect of age was unlikely to be secondary to disease duration.

Although this meta-analysis provided strong evidence of peripheral inflammatory response in ALS, and the between-study heterogeneity was partially addressed by meta-regression analyses for TNF-α studies, this meta-analysis has some limitations. First, the meta-analysis of peripheral blood levels of inflammatory cytokines in patients with ALS compared with controls provided us pooled results from case-control (cross-sectional) studies, thus it is unclear whether the increased releases of cytokines are a cause or consequence for ALS onset. Second, studies on the associations between cytokines and disease duration or disease severity are limited, therefore prevented us from assessing whether the blood inflammatory cytokines have potential to be validated biomarkers to predict disease progression and/or patient diagnosis for ALS, and future studies are necessary to address this question. Third, the limited number of studies with smaller sample sizes may have made observation of significant associations difficult for some cytokines. As examples are IL-10 and IL-17 which showed increased levels in patients with ALS, but did not research statistical significance (p = 0.081 and 0.087 respectively, see Table 1). Therefore, data from future studies on the cytokine levels in ALS that added into this meta-analysis may generate more cytokines that significantly associate with ALS. Fourth, what is the significance of the increased peripheral inflammatory response related to CNS remains unclear, thus future analyses of CNS cytokine levels in ALS are necessary to better understand the etiology of the disease. These limits highlight the need for continued investigations into the aberrations of inflammatory cytokines in ALS.

In conclusion, this meta-analysis is the first undertaken to investigate the alterations of inflammatory cytokine levels in ALS patients, and demonstrated increased peripheral blood TNF-α, TNFR1, IL-1β, IL-6, IL-8 and VEGF levels in ALS patients compared to control subjects. These results strengthen the clinical evidence of an increased inflammatory response in patients with ALS, thus providing a new insight into a potential molecular pathway that confers vulnerability to the onset and/or development of ALS, and underscores the potential role of cytokines as biomarkers for ALS, giving the robust and consistent associations between some cytokines and ALS. Therefore, more studies are needed in the future, hopefully with international cooperation, to translate the potential blood cytokine biomarkers into benefit of ALS patients.

## Electronic supplementary material

Supplementary Information
