# Supplementary material for: Increased peripheral blood inflammatory cytokine levels in amyotrophic lateral sclerosis: a meta-analysis study
Source: Sci Rep. 2017 Aug 22;7:9094. doi: 10.1038/s41598-017-09097-1 (PMC5567306; doi:10.1038/s41598-017-09097-1)
Supplement: Supplementary file 1 — Supplementary Information [file 41598_2017_9097_MOESM1_ESM.pdf]

Supplementary Information

Increased peripheral blood inflammatory cytokine levels in amyotrophic lateral sclerosis: a meta-analysis study

Yang Hu, Chang Cao, Xiao-Yan Qin , Yun Yu , Jing Yuan, Yu Zhao, Yong Cheng

Center on Translational Neuroscience, College of Life and Environmental Sciences, Minzu University of China, Beijing 100081, China

Supplementary Table: Characteristics of included studies measuring peripheral blood cytokine concentrations

Supplementary Figure 1: Sub-group analysis stratified by control type for TNF-α studies

Supplementary Figure 2: Meta-regression for TNF-α

Supplementary Table: Characteristics of included studies measuring peripheral blood cytokine concentrations

Author/year	Country	groups	N(ALS/Control)	Gender (% Male) (ALS/Control)	Age (ALS/Control)	Disease Duration (months)	Diagnosis	Sample Origin	Cytokines Measured
Babu et al. 2008	India	ALS/ NC	22/20	90.9/NA	42/43	12	El Escorial	Serum	TNF-α, IFN-γ
Baron et al. 2005	Italy	ALS/ DC	9/10	48.1/40	55.6/48.5	19.4	El Escorial	Serum	MCP-1
Balsco et al. 2016	France	ALS/NC	27/30	44.4/40	65.7/65.4	0	El Escorial	Serum	TNF-α, IL-6, IL-8, IL-10
Cereda et al. 2008	Italy	ALS/ NC	88/40	55.7/NA	61.0/NA	53	El Escorial	Plasma	TNF-α, TNFR1,
Ehrhart et al. 2015	America	ALS/ NC	13/7	92.3/42.9	53.9/57.7	41.77	El Escorial	Serum	IL-1β, IL-2, IL-4, IL-5, IL-6, IL-8, IL-10, TNF-α
Fiala et al. 2010	America	ALS/NC	32/10	50/70	57.8/56.6	24.5	El Escorial	Serum	IL-17
Furukawa et al. 2014	Japan	ALS/DC	16/15	NA/46.7	58.5/34.9	14.3	El Escorial	Serum	IL-1β, IL-2, TNF-α, TNFR1
Gupta et al. 2011	India	ALS/NC	50//50	76/78	47.4/40.0	19.0	El Escorial	Serum	VEGF, MCP-1
Gupta et al. 2012	India	ALS/ NC	44/29	77.3/79.3	47.2/38.1	19.2	El Escorial	Serum	MCP-1, VEGF
Kuhle et al. 2009	Switzerland	ALS/DC	20/20	NA	NA	9.37	El Escorial	Serum	IL-8, MCP-1
Liu et al. 2015	China	ALS/DC	52/31	69.2/61.3	52.01/49.42	19.17	El Escorial	Serum	IFN-γ

Author/year	Country	groups	N(ALS/Control)	Gender (% Male) (ALS/Control)	Age (ALS/Control)	Disease Duration (months)	Diagnosis	Sample Origin	Cytokines Measured
Lu et al. 2016	England	ALS/DC	95/88	66.3/29.5	66.8/55.9	22.4	El Escorial	Plasma	IFN-γ, TNF-α, IL-6, IL-1β, IL-2, IL-8, IL-4, IL-5, IL-10
Moreau et al. 2005	France	ALS/DC	20/20	55/80	65.95/70.2	19.15	El Escorial	Serum	IL-6, TNF-α
Ngo et al. 2015	Australia	ALS/NC	68/34	69.1/67.6	60.4/57.3	NA	El Escorial	Plasma	IL-6, IL-8, MCP-1, TNF-α
Nygren et al. 2002	Sweden	ALS/NC	13/13	46.2/46.2	57/55	41.5	El Escorial	Serum	VEGF
Oka et al. 1994	Japan	ALS/NC	15/12	NA	NA	NA	NA	Serum	ELAM-1
Poloni et al. 2000	Italy	ALS/NC	51/36	54.9/30.6	57.9/50.3	25.0	El Escorial	Serum	TNF-α, TNFR1
Rentzos et al. 2005a	Greece	ALS/DC	20/15	75/66.7	62/64	22	El Escorial	Serum	ELAM-1
Rentzos et al. 2005b	Greece	ALS/NC	16/15	62.5/60	62/60	22	El Escorial	Serum	ELAM-1
Rentzos et al. 2010	Greece	ALS/DC	22/19	68.2/52.6	58/58	17	El Escorial	Serum	IL-17
Tanaka et al. 2006	Japan	ALS/DC	37/33	51.4/69.7	59.5/56.0	19.6	El Escorial	Serum	IL-1β, IL-2, IL-4, IL-5, IL-6, IL-8, IL-10, IL-17, IFN-γ, TNF-α, MCP-1
Terenghi et al. 2006	Italy	ALS/NC	12/13	NA	NA	NA	NA	Serum	TNF-α, INF-γ, IL-2, IL-4, IL-10

Author/year	Country	groups	N(ALS/Control)	Gender (% Male) (ALS/Control)	Age (ALS/Control)	Disease Duration (months)	Diagnosis	Sample Origin	Cytokines Measured
Vlam et al. 2015	Netherlands	ALS/NC	25/50	72/88	68/53	24	NA	Serum	IL-2, IL-6, TNF-α,
Wilms et al. 2003	Germany	ALS/DC	29/11	NA	61.6/56.8	8	El Escorial	Serum	MCP-1
Zhang et al. 2006	America	ALS/NC	42/38	64.3/63.2	60.0/58.3	24.0	El Escorial	Plasma	MCP-1

Abbreviations: ALS, amyotrophic lateral sclerosis; N, sample size; DC, disease control; NC: normal control; TNF, tumor necrosis factor-α; TNFR1, TNF receptor 1; IFN, interferon; IL, interleukin; ELAM, endothelial leukocyte adhesion molecule; MCP-1, Monocyte chemotactic protein-1; VEGF, vascular endothelial growth factor

Supplementary Figure 1

Sub-group analysis stratified by control type for TNF-α

Group by Control type	Study name	Statistics for each study			
		Hedges's g	Lower limit	Upper limit	p-Value
DC	Furukawa et al. 2014	0.003	-0.683	0.689	0.992
DC	Lu et al. 2016	0.497	0.204	0.791	0.001
DC	Moreau et al. 2005	0.000	-0.607	0.607	1.000
DC	Tanaka et al. 2006	0.000	-0.464	0.464	1.000
DC		0.201	-0.106	0.509	0.200
NC	Babu et al. 2008	2.200	1.442	2.958	0.000
NC	Balsco et al. 2016	0.706	-0.183	1.595	0.120
NC	Cereda et al. 2008	0.907	0.519	1.295	0.000
NC	Ehrhart et al. 2015	0.049	-0.831	0.929	0.913
NC	Ngo et al. 2015	0.425	0.013	0.838	0.043
NC	Poloni et al. 2000	0.912	0.468	1.356	0.000
NC	Terenghi et al. 2006	3.460	2.237	4.682	0.000
NC	Vlam et al. 2015	0.000	-0.475	0.475	1.000
NC		0.972	0.430	1.514	0.000

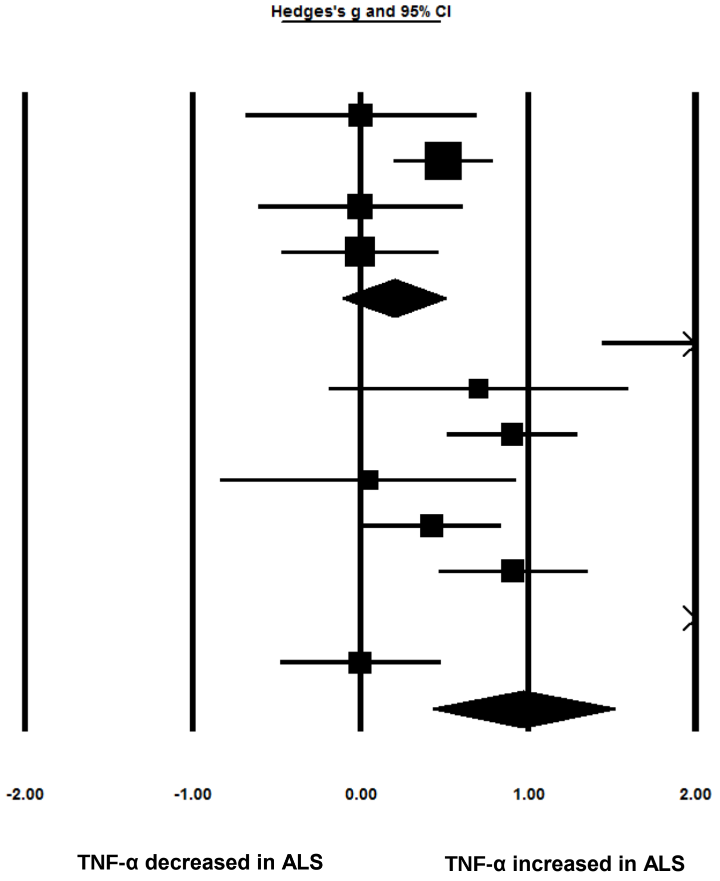

Forrest plot showing pooled results comparing blood TNF-α levels between ALS patients and control subjects stratified by control type (disease and normal control). The sizes of the squares are proportional to study weights.

Supplementary Figure 2

Meta-regression for TNF-α

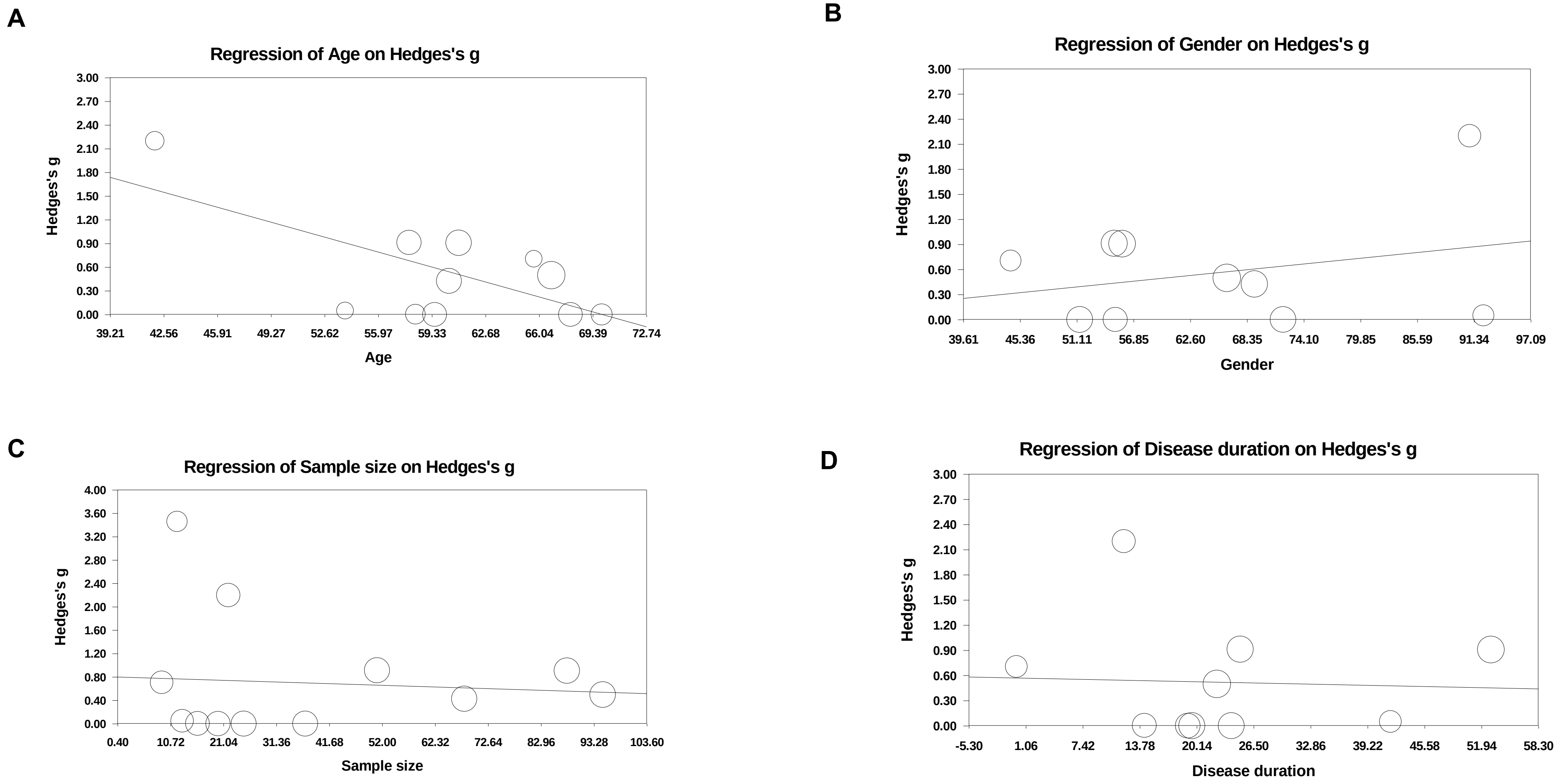

Association between age **(A)**, gender **(B)**, sample size **(C)**, disease duration **(D)** and effective size (Hedges's g) for TNF-α studies. The sizes of the circles are proportional to study weights.
